# Effect of Iliotibial Band Myofascial Release Combined with Valgus Correction Exercise on Pain, Range of Motion, Balance, and Quality of Life in Patients with Grade II Knee Osteoarthritis: A Randomized Clinical Trial

**DOI:** 10.3390/life14111379

**Published:** 2024-10-27

**Authors:** Mubashra Nouman, Javeria Shabnam, Sahreen Anwar, Wajida Perveen, Dan Iulian Alexe, Rubén Sánchez-Gómez, Mihai Adrian Sava, Cristina Ioana Alexe

**Affiliations:** 1 Department of Physical Therapy, Riphah International University, Islamabad 45320, Pakistan; mubasharanouman@gmail.com (M.N.); javeriarana1998@gmail.com (J.S.); 2 University Institute of Physical Therapy, The University of Lahore, Lahore 54570, Pakistan; sahreenanwar@yahoo.com; 3 CMH Lahore Medical College & IOD (NUMS Rawalpindi), Lahore Cantt, Lahore 54810, Pakistan; wajida_perveen@cmhlahore.edu.pk; 4 Department of Physical and Occupational Therapy, “Vasile Alecsandri” University of Bacău, 600115 Bacău, Romania; 5 Instituto de Investigación Sanitaria, (IdISSC) Hospital Clínico San Carlos, 28040 Madrid, Spain; rusanc02@ucm.es; 6 Faculty of Nursing, Physiotherapy and Podiatry, Department of Nursing, University Complutense of Madrid, 28040 Madrid, Spain; 7 Department of Physical Education and Sports Performance, “Vasile Alecsandri” University of Bacău, 600115 Bacău, Romania; sava.adrian@ub.ro

**Keywords:** exercises, iliotibial, knee osteoarthritis, myofascial release, valgus knee deformity

## Abstract

The objective of this study was to find out the effect of the myofascial release technique combined with valgus correction exercise on the pain, range of motion, balance, and quality of life in participants with grade II knee osteoarthritis. Forty participants with grade II knee osteoarthritis were randomly assigned into two treatment groups in the present clinical trial; group A was the myofascial release group, and group B was the myofascial release with valgus correction exercises group. Measurement included pain, balance, range of motion, and quality of life, as measured through the visual analog scale (VAS), Berg balance scale (BBS), goniometer, and knee injury and osteoarthritis outcome score (KOOS), respectively. The data were collected at the baseline and 3rd and 6th weeks. The between-groups comparison at the end of the 6th week showed significant results in the iliotibial band myofascial release with the valgus correction exercise group (*p* < 0.001). The within-group difference showed improvement in both groups individually, with more significant values in group B. The current study showed that the myofascial release combined with valgus correction exercises can effectively improve the pain, range of motion, balance, and quality of life in participants with grade II knee osteoarthritis. Trial Registration: IRCT20230216057434N3.

## 1. Introduction

Knee osteoarthritis is a degenerative disorder characterized by the gradual deterioration of the articular cartilage and underlying subchondral bone within the knee joints [1]. Based on Disability Adjusted Life Year (DALY) estimates, osteoarthritis is the second most prevalent musculoskeletal disorder among the elderly, primarily affecting weight-bearing joints, such as the knees, hips, and feet [2]. The incidence of knee osteoarthritis has observed a substantial rise in recent decades and it holds the 10th position among the leading causes of disability [3]. The development of knee osteoarthritis is influenced by a combination of risk factors, including advanced age, previous knee injuries, high body mass index, joint misalignment or instability, hormonal changes, genetic mutations, and local biomechanical factors [2]. The interaction between chronic pain and depressive episodes gives rise to a detrimental cycle, where pain restricts physical activity, which subsequently contributes to amplified knee pain and weight gain [4].

Valgus knee is a sub-categorization of knee osteoarthritis that refers to a misalignment of the lower extremity where the mechanical axis is located laterally to the center of the knee joint [5]. This configuration anomaly has been associated with an increased vulnerability to chondral damage resulting from modified joint loading. Additionally, it raises the likelihood of developing valgus knee osteoarthritis at an earlier stage and experiencing a more rapid disease progression [6]. Valgus knee deformity, although constituting a minority, accounting for only 10% of total knee arthroplasties (TKAs), is commonly considered the most challenging condition to address and manage effectively [7]. Giving precedence to self-management techniques, exercise, and physical activity is crucial as a primary approach to manage the condition effectively [8]. Physical therapy interventions involve various techniques, including therapeutic exercises, taping, manual therapy, and patient education. It is of utmost importance to establish comprehensive guidelines for the treatment of osteoarthritis (OA) and associated deformities [9].

Myofascial release (MFR) therapy is a manual therapy technique developed by Barnes that involves the application of a low-load, long-duration stretch to the myofascial complex. It helps to reduce the restrictive barriers or fibrous adhesions seen between the layers of fascial tissues to restore an optimal length, decrease pain, and improve function [10]. Osteoarthritis of the knee involves tightening of the iliotibial band and shortening of the hamstring muscles, and myofascial release is a frequently utilized technique that has demonstrated efficacy in mitigating symptoms associated with it [11]. When used in conjunction with other conventional therapies, myofascial release has exhibited its effectiveness in delivering rapid pain relief and diminishing tissue tenderness [12]. According to a study conducted by H Kim and W Shin to determine the immediate effect of the pressure pain threshold and flexibility in the tensor fascia latae and iliotibial band using various foam roller exercise methods, static self-myofascial release showed a significant difference in pain threshold as compared with another intervention group [13].

Evidence suggests that apart from conservative measures, engaging in exercises that target muscle strengthening and balance can be advantageous in mitigating the risk of progression of knee osteoarthritis. In a study, it was observed that there is a strong association between increased knee valgus and excessive lateral torso lean toward the same side [14]. Valgus correction exercises are focused exercises that target muscle strengthening and realignment of the lower limb, as insufficient neuromuscular control in the proximal segments, particularly the hip, is believed to contribute to the heightened susceptibility to injury. Consequently, enhancing neuromuscular control through various exercise protocols has the potential to significantly enhance biomechanical profiles [15]. Integrating diverse exercise modalities that prioritize precise movement control and optimal alignment of the limbs and torso effectively mitigates the occurrence of knee valgus, consequently lowering the risk of injury within athletic populations [16]. In scientific terms, the application of the VCEs (valgus correction exercises) program has the potential for notable enhancements in various aspects among individuals with grade II knee osteoarthritis.

The primary aim of our study was to evaluate the effect of myofascial release combined with valgus correction exercises (VCEs) in grade Ⅱ knee osteoarthritis participants with iliotibial band tightness and valgus knee deformity. Critical appraisal of the recent reviews aimed to improve the quality and reliability of future works in this field [17].

## 2. Materials and Methods

This was a single-blinded, parallel-group, randomized clinical trial conducted in the outpatient Physical Therapy Department of Allied Hospital, Faisalabad, Pakistan, from 14 February 2023 to 17 July 2023. The trial was conducted based on the principles of the Helsinki Declaration and was documented according to the Consolidated Standards of Reporting Trials (CONSORT) statement (Supplementary Materials).

The ethical approval was obtained from the Ethical Review Committee of Riphah International University, Faisalabad, Pakistan (RCRAHS/REC/08). After the initial screening, consent was given by participants to participate in this study.

### 2.1. Sampling and Randomization

**Figure 1:**
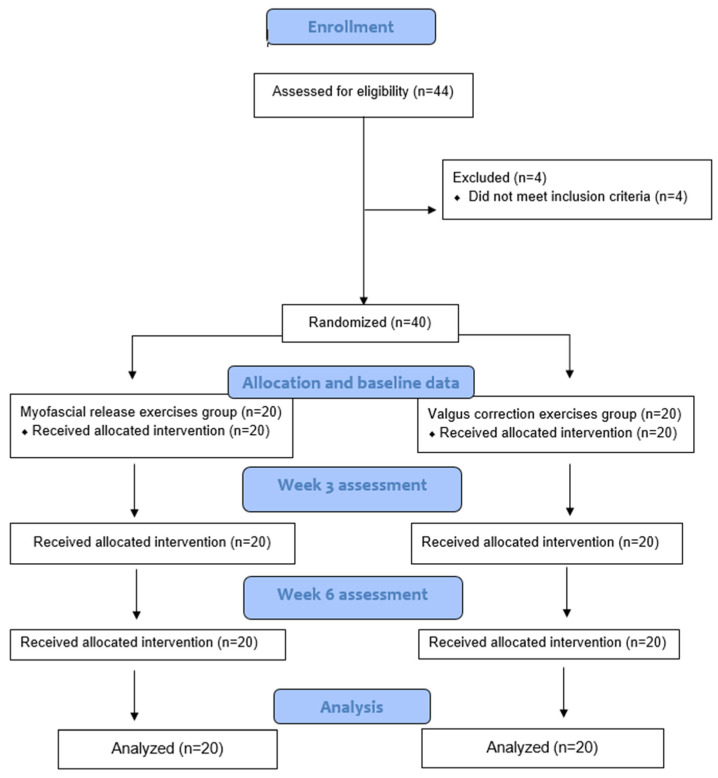
CONSORT chart.

The sample size was calculated using the Giga calculator with an 80% power of study, 5% confidence interval, and 5% margin of error. The calculated sample size was 40; after adding a 10% attrition rate, the final sample size was 44. Participants were selected through a non-probability purposive sampling technique. The primary outcome was pain and a difference of 1 point on a scale of 0–10 (VAS) was considered clinically significant. The patients with grade II knee osteoarthritis that presented to the Outpatient Department of Allied Hospital were screened according to the inclusion criteria to be included in this study. The inclusion criteria for the selection of the participants were as follows: (1) healthy women and men aged between 40 and 60 years old and (2) grade II knee osteoarthritis diagnosed as per the ACR (American College of Rheumatology) criteria [18]. The exclusion criteria for the participants were as follows: (1) having any pain and/or any lower limb or foot injuries at the time of the test or 1 year ago, (2) having any restriction of the range of movement on a lower limb, and (3) being under any medication effects at the time of the test. The participants were divided into 2 groups, where group A used the myofascial release technique and group B used the myofascial release technique with valgus correction exercises, through a Random allocation computer software version 2 (Figure 1). The software was used to assign the total sample size into two groups using automated randomization, taking into account an attrition rate. Both groups were kept unaware of the instructions given to the other group. The outcome assessor, an experienced therapist, was blinded to the treatment allocation.

### 2.2. Intervention

Participants in group A were directed to lie on their side with the affected side facing upward. They received a heat pack on the outer side of the thigh (iliotibial area) and then underwent fifteen minutes of Transcutaneous Electric Nerve Stimulation that targeted the iliotibial band. After the TENS application, the participants were instructed to perform two sets of quadriceps isometric exercises that involved 10 repetitions each, with a hold of 10 s.

After undergoing the exercise therapy, the myofascial release technique involved identifying and applying gradually increasing pressure to trigger points using a therapist’s thumb until the fascia was released. The therapist applied pressure on the affected side for 30 s to release each trigger point and released the pressure as the tension decreased. The total treatment duration was 40 min and sessions occurred twice weekly over 6 weeks, which totaled 12 sessions. The group B participants received the same initial treatment as group A, followed by ten minutes of corrective exercises for valgus. The details of the valgus correction exercises are as follows.

### 2.3. Valgus Correction Exercises (VCEs)

The valgus correction exercises (VCEs) method based on the approaches of Prentice and Rabelo was followed in this study [19]. To control the movement of the pelvis and knee in the frontal plane, verbal and visual feedback methods were used with the help of a mirror. Before starting the exercises, the correct and incorrect ways of performing each exercise were shown to the participants. Throughout the exercises, the participants were instructed to control their pelvic and knee movements, with guidance like “keep your knees toward the toes”, “stop your knees from rotating internally”, and “keep the pelvis at a symmetric level”. The examiner provided verbal feedback only at the beginning of each training session but repeated it if the patient did not maintain the correct position [20]. The exercises were performed in the following sequence:

(a) Squat in front of the mirror: 0–60° of knee flexion, performed in front of the mirror to ensure the knee did not exceed the midfoot. Three sets of 12 repetitions, with a 10 s isometric contraction. The tendency to use the active valgus strategy during squats was removed.

(b) Lateral walk with elastic resistance around the forefoot: 3 sets of 12 repetitions. This improved the hip abductor, hamstring, and gluteus strength to reduce medial knee displacement.

(c) Hip lateral rotation: 3 sets of 12 repetitions. This improved the range of motion of the hip adductors and hip internal rotators.

### 2.4. Data Collection Procedure

The data were collected at the baseline, in the 3rd week, and in the 6th week of the treatment. The outcome measures were pain, balance, range of motion, and quality of life. The pain was assessed by a visual analog scale (VAS), a reliable tool that comprises a line that is 10 cm long, featuring two endpoints indicating 0 (indicating “no pain”) and 10 (representing “pain at the worst possible level”) [21]. Balance was assessed by the Berg balance scale (BBS), a reliable tool for measuring static balance [22]. The range of motion of the knee was calculated by a universal goniometer, a frequently used reliable and valid instrument [23]. The knee injury osteoarthritis outcome score (KOOS) was used to assess the pain, symptoms, activities of daily living, and quality of life. The KOOS is a patient-reported outcome measure that evaluates the patient’s perspective regarding the health, symptoms, and functionality of their knee [24].

### 2.5. Statistical Analysis

The data were analyzed using the SPSS version 26.0 (SPSS Inc., Chicago, IL, USA). The normality of the data was tested by using Shapiro–Wilk’s test. As the data were normally distributed, a parametric mixed-model ANOVA was applied to see the between-groups differences at three different time points. For the within-group differences, pairwise comparisons with paired *t*-tests were used.

## 3. Results

During the six weeks of the intervention program, all 40 participants completed this study. There were no significant differences in the baseline mean values of the demographics in both groups. The mean ages, heights, and weights of the control and VCE groups and the standard deviations are shown in Table 1.

The results of the mixed-model ANOVA for the comparison of the mean scores for the visual analog scale at different time points (3rd and 6th weeks) revealed a statistically significant difference. At the baseline, the mean VAS (SD) scores for the myofascial and valgus correction exercise groups were 6.500 (1.469) and 6.050 (1.276), respectively. In myofascial group there was an improvement in pain with the mean VAS score, 4.4500 (0.998) and 4.750 (1.293) at 3rd and 6th week respectively whereas in valgus correction exercises group the improvement was 3.250 (0.786), and 2.300 (0.470) at 3rd and 6th week respectively (Table 2).

The results of the mixed-model ANOVA for knee flexion and extension ROM revealed a significant difference for different time points. For the knee flexion ROM, the baseline mean (SD) values for the myofascial group and valgus correction exercises group were 103.48 (20.799) and 93.00 (17.237), respectively. At the 3rd and 6th weeks, the mean (SD) values were 113.47 (20.798) and 103.00 (17.236), respectively, for the myofascial group and 122.85 (8.928) and 125.30 (4.996), respectively, for the valgus correction exercises group. Both groups from the baseline to the 3rd week showed a mean difference of 9.225, with a significant *p*-value (<0.001), and from the 3rd week to the 6th week, showed a mean difference of 16.612, with a significant *p*-value (<0.001).

For the knee extension ROM, the baseline mean (SD) values for the myofascial group and valgus correction exercises group were −5.55 (1.32) and −4.05 (2.743), respectively. At the 3rd and 6th weeks, the mean (SD) values were −5.200 (1.151) and 4.20 (1.765), respectively, for the myofascial group and −7.32 (2.002) and −5.65 (2.581), respectively, for the valgus correction exercises group. Both groups from the baseline to the 3rd week showed a mean difference of 0.100, with a non-significant *p*-value (0.998), and from the 3rd week to the 6th week showed a mean difference of 1.687, with a significant *p*-value (<0.001) (Table 3).

The results of the mixed-model ANOVA of the Berg balance scale revealed a significant difference between different time points. The baseline mean (SD) values for the myofascial and valgus correction exercise groups were 37.700 (5.130) and 36.800 (5.473), respectively. At the 3rd and 6th weeks, the mean (SD) values were 42.700 (3.881) and 42.800 (5.492), respectively, for the myofascial group and 43.00 (4.633) and 46.200 (4.674), respectively, for the valgus correction exercises group. Both groups from the baseline to the 3rd week showed a mean difference of 1.850, with a non-significant *p*-value (0.069), and from the 3rd week to the 6th week showed a mean difference of 5.500, with a significant *p*-value (<0.001).

For the knee injury and osteoarthritis outcome score (KOOS), the baseline mean (SD) of the myofascial group and valgus correction exercises group were 56.270 (95.401) and 55.675 (5.111), respectively. At the 3rd and 6th weeks, the mean (SD) values were 6.770 (5.401) and 63.255 (4.611), respectively, for the myofascial group and 81.100 (6.820) and 82.600 (2.036), respectively, for the valgus correction exercises group. Both groups from the baseline to the 3rd week showed a mean difference of 8.550, with a non-significant *p*-value (0.071), and from the baseline to the 6th week showed a mean difference of 25.878, with a significant *p*-value (<0.001) (Table 4).

For the within-group comparison, a multi-repeated ANOVA test was applied. At the baseline, the mean (SD) values of the knee flexion for the myofascial group were 103.48 (20.799), at the 3rd week 111.925 (20.844), and at the 6th week 122.85 (8.928), with an F-value of 42.176 and a significant *p*-value <0.001. For the knee extension, the mean (SD) values at the baseline were −5.55 (1.932), at the 3rd week −5.200 (1.51), and at the 6th week −7.32 (2.002), with an F-value of 10.318 and a *p*-value <0.001 (Table 5).

The multi-repeated ANOVA for the Berg balance scale showed a mean (SD) of 36.80 (5.473) at the baseline, at the 3rd week 42.800 (5.492), and at the 6th week 46.20 (4.674), with an F-value of 933.164 and a *p*-value < 0.001. For the knee injury and osteoarthritis outcome scores, the mean (SD) was 55.675 (5.111) at the baseline, 63.255 (4.611) at the 3rd week, and 82.600 (2.036) at the 6th week, with an F-value of 230.046 and a *p*-value < 0.001 (Table 6).

## 4. Discussion

This randomized controlled trial investigated the effect of the iliotibial band myofascial release combined with valgus correction exercises on the pain, range of motion, balance, and quality of life in participants with grade II knee osteoarthritis. The trial illustrated that the participants who underwent the iliotibial band myofascial release alongside valgus correction exercise displayed more substantial outcomes than those who only received the iliotibial myofascial release. Knee osteoarthritis is often linked with pain, restricted range of motion, and diminished joint function. Apart from other conservative options, exercise therapy is one of the most established techniques, and mainly focuses on improving muscle strength, joint mobility, and stability [25].

The results of this study are consistent with a study by Jafarnezhadgero et al., in which the effects of a 16-week corrective training program on three-dimensional joint moments of the dominant limb in kids with genu varus were observed. The overall study showed significant improvement in all ranges regardless of the dominant and non-dominant lower limb [26]. The current study showed that combining myofascial release with the valgus correction exercise significantly improved all knee ROM measurements (*p* < 0.001).

According to the scientific literature, incorporating VCI (valgus correction instruction exercise) training as a supplementary approach may have potential benefits for the prevention and treatment of lower extremity injury in participants with patellofemoral pain syndrome (PFPS) [20]. These improvements included enhanced performance, reduction in the knee dynamic valgus angle, and increased strength in the participants. In our study, the hip and knee corrective exercises resulted in an improved balance range of motion and quality of life, as evidenced by the VCE group, which showed that participants involved in the group that performed valgus corrective exercises in combination with the IT band MR illustrated more significant results as compared with the group that received only the IT band MR (*p* < 0.01). The results of this study are supported by another study on the effects of National Academy of Sports Medicine (NASM) exercise in retired athletes and it was observed that NASM exercise demonstrated significant improvement in the ROM, Berg balance scale score, and dynamic gait pattern (*p* < 0.05) [27].

In another study to see the effects of weight-bearing and non-weight-bearing exercises in participants with osteoarthritis of the knee, it was observed that pain, balance, and proprioception were improved in the weight-bearing group [28]. The valgus correction regime used in our study advocates for the same effects while directing the weight bearing in the optimal position of an arthritic knee joint. In a narrative review, it was concluded that closed kinetic chain exercises are effective in improving the pain, range of motion, and quality of life in participants with knee osteoarthritis [29]. The valgus correction exercises involve weight bearing in a closed chain position with a resistance band or a medicine ball and augment the results of this review, as the pain, range of motion, and KOOS were improved.

According to a systematic review and meta-analysis to see the effects of exercise therapy and biomechanical loads during walking, it was found that exercise therapy combined with biomechanical interventions, such as valgus correction, improved the pain and strength in participants with knee osteoarthritis [30]. In our study, we did not measure the improvement in the strength of various muscles involved in valgus correction exercises, but an improvement in pain was observed in the VCE group.

This study investigated a novel approach that involved the integration of iliotibial band myofascial release and valgus correction exercise as combined interventions.

This unique combination has the potential to effectively target various aspects of knee osteoarthritis symptoms, such as pain management, improved range of motion, enhanced balance, and overall quality of life. By simultaneously addressing multiple dimensions of the condition, this innovative approach holds promise in providing comprehensive relief and improving the well-being of individuals affected by knee osteoarthritis.

### Limitations

The present study had certain limitations that should be acknowledged. First, the exercise program implemented in this research was conducted over a relatively short duration of 6 weeks, which prevented the assessment of long-term effects. Second, the absence of a placebo control group was another significant limitation. The sample size in the current study was relatively small, potentially impacting the generalizability of the findings. To improve the external validity, it is recommended to conduct future studies with larger and more diverse samples.

## 5. Conclusions

The current study concluded that the iliotibial band myofascial release combined with valgus correction exercises was effective in improving the pain, balance, range of motion, and quality of life in grade II knee osteoarthritis patients. This unique combination has the potential to contribute to conventional osteoarthritis rehabilitation programs.

## Figures and Tables

**Table 1:** Demographic characteristics of participants (n = 40).

	Mean (SD)	Mean (SD)	*p*-Value
	Myofascial Group (n = 20)	VCE Group (n = 20)	
Age, Y	51.050 (6.425)	52.143 (5.125)	0.521
Height, centimeters	159.71 (0.392)	158.62 (0.8120)	0.613
Weight, kg	73.675 (8.065)	74.123 (7.014)	0.512

Y—years.

**Table 2:** Between-groups comparison of visual analog scale at baseline, 3rd week, and 6th week.

	Groups	Descriptive Statistics			
		N	Baseline	At 3rd Week	At 6th Week
			Mean (SD)	Mean (SD)	Mean (SD)
Visual analog scale	Myofascial group	20	6.500 (1.469)	4.450 (0.998)	4.750 (1.293)
	Valgus correction exercises group	20	6.050 (1.276)	3.250 (0.786)	2.300 (0.470)
	Mixed-Model ANOVA (Pairwise Comparison)				
			Mean Difference	Std. Error	*p*-Value
	Baseline	20	−0.325	0.207	0.374
	3rd week	20	−1.925	0.167	<0.001
	3rd–6th week	20	−2.250	0.272	<0.001

SD—standard deviation, Std—standard error.

**Table 3:** Between-groups comparison of knee flexion and extension ROM at baseline, 3rd week, and 6th week.

	Groups	Descriptive Statistics			
		N	Baseline	At 3rd Week	At 6th Week
			Mean (SD)	Mean (SD)	Mean (SD)
Knee flexion	Myofascial groups	20	103.48 (20.799)	113.47 (20.798)	122.85 (8.9284)
	Valgus correction exercises groups	20	93.00 (17.237)	103.00 (17.236)	125.30 (4.996)
	Mixed-Model ANOVA (Pairwise Comparison)				
		N	Mean Difference	Std. Error	*p*-Value
	Baseline–3rd week	20	9.255	0.475	<0.001
	3rd–6th week	20	16.612	3.052	<0.001
	Baseline–6th week	20	25.837	3.053	<0.001
Knee extension	Groups	N	Baseline	At 3rd week	At 6th week
			Mean (SD)	Mean (SD)	Mean (SD)
	Myofascial group	20	−5.55 (1.932)	−5.20 (1.151)	−7.32 (2.002)
	Valgus correction exercise group	20	−4.05 (2.74)	−4.20 (1.765)	−5.65 (2.581)
	Mixed-Model ANOVA (Pairwise Comparison)				
			Mean Difference	Std. Error	*p*-Value
	Baseline–3rd week	20	0.100	0.296	0.998
	3rd week–6th week	20	1.687	0.385	<0.001
	Baseline–6th week	20	1.788	0.278	<0.001

SD—standard deviation, Std—standard error.

**Table 4:** Between-groups comparison of Berg balance scale and KOOS at baseline, 3rd week, and 6th week.

	Groups		Descriptive Statistics		
		N	Baseline	At 3rd Week	At 6th Week
			Mean (SD)	Mean (SD)	Mean (SD)
Berg balance scale	Myofascial group	20	37.70 (5.130)	42.70 (3.88)	43.00 (4.633)
	Valgus correction exercise group	20	36.80 (5.473)	42.800 (5.492)	46.200 (4.674)
	Mixed-Model ANOVA (Pairwise Comparison)				
		N	Mean Difference	Std. Error	*p*-Value
	Baseline–3rd week	20	1.850	0.561	0.069
	3rd week–6th week	20	5.500	0.232	<0.001
	Baseline–6th week	20	7.350	0.539	<0.001
Knee injury and osteoarthritis outcome score	Groups	N	Baseline	At 3rd Week	At 6th Week
			Mean (SD)	Mean (SD)	Mean (SD)
	Myofascial group	20	56.270 (5.401)	65.770 (5.401)	81.100 (6.820)
	VCE group	20	55.675 (5.111)	63.255 (4.611)	82.600 (2.036)
	Mixed-Model ANOVA (Pairwise Comparison)				
		N	Mean Difference	Std. Error	*p*-Value
	Baseline–3rd week	20	8.540	0.288	0.071
	3rd week–6th week	20	25.878	1.164	<0.001
	Baseline–6th week	20	17.337	1.130	<0.001

SD—standard deviation, Std—standard error.

**Table 5:** Within-group differences for visual analog scale, knee flexion, and knee extension.

		Descriptive Statistics			Repeated-Measure ANOVA	
Treatment Groups	Outcome		N	Mean (SD)	F	*p*-Value
Myofascial	VAS	Baseline	20	6.500 (1.469)	42.736	<0.001
		At 3rd week	20	4.750 (0.998)		
		At 6th week	20	4.750 (1.293)		
Valgus correction exercises		Baseline	20	6.050 (1.276)	40.474	<0.001
		At 3rd week	20	3.250 (0.786)		
		At 6th week	20	2.300 (0.470)		
Myofascial	Knee flexion	Baseline	20	103.48 (20.799)	42.176	<0.001
		At 3rd week	20	111.925 (20.844)		
		At 6th week	20	122.85 (8.928)		
Valgus correction exercises		Baseline	20	93.00 (17.237)	72.941	<0.001
		At 3rd week	20	103.00 (17.236)		
		At 6th week	20	125.30 (4.996)		
Myofascial	Knee extension	Baseline	20	−5.55 (1.932)	10.318	<0.001
		At 3rd week	20	−5.200 (1.151)		
		At 6th week	20	−7.32 (2.002)		
Valgus correction exercises		Baseline	20	−4.05 (2.743)	9.717	<0.001
		At 3rd week	20	−4.200 (1.765)		
		At 6th week	20	−5.65 (2.581)		

SD—standard deviation.

**Table 6:** Within-group differences for Berg balance scale and KOOS.

		Descriptive Statistics n = 20			Repeated-Measure ANOVA	
Treatment Groups		Time Points	N	Mean (SD)	F	*p*-Value
Myofascial	Berg balance scale	Baseline	20	36.800 (5.473)	933.164	<0.001
		At 3rd week	20	42.800 (5.492)		
		At 6th week	20	46.200 (4.672)		
Valgus correction exercises		Baseline	20	37.700 (5.130)	83.436	<0.001
		At 3rd week	20	42.700 (3.881)		
		At 6th week	20	43.000 (4.633)		
Myofascial	KOOS	Baseline	20	55.675 (5.111)	230.046	<0.001
		At 3rd week	20	63.255 (4.611)		
		At 6th week	20	82.600 (2.036)		
Valgus correction exercises		Baseline	20	56.270 (5.401)	159.074	<0.001
		At 3rd week	20	65.770 (5.401)		
		At 6th week	20	81.100 (6.820)		

SD—standard deviation.

## Data Availability

The raw data supporting the conclusions of this article will be made available by the authors on request.

## Supplementary Materials

The following supporting information can be downloaded at: https://www.mdpi.com/article/10.3390/life14111379/s1, CONSORT 2010 checklist of information to include when reporting a randomised trial.
